# SOCIAL ISOLATION AND LONELINESS AMONG OLDER ADULTS IN SUBSIDIZED SENIOR HOUSING: DOES IMMIGRATION STATUS MATTER?

**DOI:** 10.1093/geroni/igac059.1698

**Published:** 2022-12-20

**Authors:** Chung Hyeon Jeong, Jihye Baek, ByeongJu Ryu, Jina Park, BoRin Kim, Sojung Park

**Affiliations:** University of New Hampshire, Durham, New Hampshire, United States; Washington University in St. Louis, St. Louis, Missouri, United States; Chuncheon Hyoja Social Welfare Center, Chuncheon-si, Kangwon-do, Republic of Korea; Washington University in St. Louis, St. Louis, Missouri, United States; University of New Hampshire, Durham, New Hampshire, United States; Washington University in St. Louis, St. Louis, Missouri, United States

## Abstract

Older immigrants are at increased risk for social isolation and loneliness due to cultural and linguistic barrier to forming social networks. The vulnerability to social isolation could be exacerbated by the gaps between older immigrants’ cultural expectations on social relations and actual social connectivity. Guided by the cognitive discrepancy model of loneliness, this study examined how social isolation influences loneliness among low-income older residents living in subsidized senior housing and if the relation varies by immigration status. Survey data were collected in 2019 and 2020 from 231 residents in subsidized senior housing communities located in St. Louis and Chicago. Social isolation was measured by frequency of social contacts, accounting for the types of relationships (family vs. friends) and the modes of contacts (in-person vs. remote). Loneliness was measured by the Revised University of California Los Angeles loneliness scale. Perceived quality of friendship was also included in the analytic models. The results of multiple linear regression showed that having positive friendship (b=-1.00, p<.001) and more frequent in-person contacts with friends (b=-1.29, p<.001) were negatively associated with loneliness, respectively. A significant moderating effect was found that older immigrants felt less lonely than non-immigrant counterparts when they had more in-person family contacts (b=-1.09, p<.05). The results suggest that the impacts of social isolation on loneliness among senior housing residents could differ depending on immigration status. The findings of this study could contribute to developing culturally sensitive interventions to enhance social connectedness and reduce loneliness among older immigrants in subsidized senior housing.

